# Oxidized albumin and its association with mortality in critically ill Covid-19 patients: a retrospective cohort study

**DOI:** 10.1186/s40635-026-00872-x

**Published:** 2026-03-02

**Authors:** Teun E.M. Aben, Johan Helleberg, Jonathan Grip, Olav Rooyackers

**Affiliations:** 1 Department of Clinical Science Intervention and Technology (CLINTEC), Division of Anaesthesiology and Intensive Care, Karolinska Institutet, Huddinge, Sweden; 2 Department of Perioperative Medicine and Intensive Care, Karolinska University Hospital, Huddinge, Sweden

**Keywords:** Oxidized albumin, Covid-19, Critical illness, Oxidative stress, Hospital mortality

## Abstract

**Background:**

Albumin is the most abundant protein in the human circulation and has many important functions. Recent studies have shown that albumin is a free radical scavenger and can be oxidized to single (HNA-1) or double (HNA-2) oxidized albumin. Oxidized albumin is a predictor for mortality in liver disease, but little is known about oxidized albumin in other diseases. This study aims to explore oxidized albumin levels in critically ill Covid-19 patients and its association with hospital mortality.

**Methods:**

In this single-center, retrospective cohort study we included Covid-19 patients (*n* = 164) treated on the ICU of Karolinska University Hospital between April 2020 and May 2021. Patient data were gathered from the electronic patient records. Oxidized albumin fractions were measured in plasma samples collected within the first 48 h of ICU admission and compared with healthy volunteers (*n* = 10). To assess the clinical relevance of oxidized albumin, descriptive statistics were performed after dividing the study group in three tertiles based on HNA-1 levels and two groups based on the presence and absence of HNA-2. A post hoc multivariable linear regression analysis was performed to assess the correlation between oxidized albumin fraction and creatinine levels.

**Results:**

HNA-1 levels were 5.1 percent point higher (*p* = 0.01) in Covid-19 patients than in healthy controls. There was no significant difference in HNA-2 levels. Hospital mortality, length of ICU stay and duration of mechanical ventilation did not differ significantly between patients with high levels of oxidized albumin and patients with low levels of oxidized albumin. Creatinine levels and sequential organ failure assessment (SOFA) scores were higher in patients with more oxidized albumin. Multivariable linear regression showed a weak but clinically relevant correlation between the fraction of oxidized albumin and creatinine, when corrected for age and chronic kidney disease before ICU admission (*R*^2^ 0.31, *p* < 0.001).

**Conclusion:**

Fractions of HNA-1 were higher in Covid-19 patients compared to healthy controls. In critically ill Covid-19 patients elevated levels of oxidized albumin were not associated with higher hospital mortality. Higher HNA-1 levels were associated with higher creatinine levels and higher SOFA scores. These findings contribute to increased knowledge about oxidized albumin in critically ill Covid-19 patients and can inspire future research.

**Supplementary Information:**

The online version contains supplementary material available at 10.1186/s40635-026-00872-x.

## Background

Albumin is one of the most versatile proteins in the human circulation. It plays a major role in maintaining oncotic pressure due to its molecular mass and negative charge [1, 2]. Other functions of albumin include the transport of metabolites, hormones, fatty acids, peptides, essential metal ions and pharmaceuticals [3, 4]. Finally, albumin has a (pseudo)esterase activity and contributes to the breakdown of pharmaceuticals, for example acetylsalicylic acid [3].

Recent studies have shown that albumin can also act as an antioxidant. Albumin oxidation occurs mostly on the Cysteine-34 (Cys-34) amino acid and is closely related to oxidative stress [1, 5, 6]. In a state of oxidative stress, levels of reactive oxygen species and other free radicals are high [7]. Albumin reacts with these free radicals, which leads to the formation of oxidized albumin [8].

Three different forms of oxidized albumin can be identified based on the redox state of the Cys-34 amino acid [1, 2]. In a physiological state, 70 to 80% of the albumin in the human plasma is non-oxidized, also known human mercaptalbumin (HMA) [1, 4, 5]. Single oxidation of Cys-34 leads to Human Non-Mercaptalbumin-1 (HNA-1) which can be reduced to HMA. The HNA-1 variant makes up for the rest of the albumin in healthy individuals, which is approximately 20 to 30% of plasma albumin [1, 4, 5]. Double-oxidation of albumin leads to a non-reducible form called Human Non-Mecaptalbumin-2 (HNA-2). In healthy individuals, HNA-2 is present in concentrations lower than 5% or even absent [1, 2, 6, 8].

Increased levels of oxidized albumin have been detected in several diseases. For example, in patients with diabetes, liver failure or kidney failure [1, 4, 6]. In all these diseases, oxidative stress is an important component of the pathophysiology. Oxidative stress is common in both patients treated in the Intensive Care Unit (ICU), for example with sepsis, and patients with Covid-19 [2, 9–12]. Besides, the delivery of high amounts of oxygen or hyperoxia in mechanically ventilated patients can contribute to the formation of free oxygen radicals and oxidative stress [13, 14].

Sparse research on the clinical implications of oxidized albumin has resulted in promising insights. Double-oxidized albumin (HNA-2) is an accurate prognostic factor for 30- and 90-day mortality in liver cirrhosis patients with acute decompensation, it performs as well as the Model of End stage Liver Disease (MELD) score [2, 9]. For patients with kidney disease, the presence of oxidized albumin is associated with higher mortality and higher rates of cardiovascular disease [15–17]. Oxidized albumin can also be used to predict progression of kidney disease, but the prognostic accuracy is relatively low [15]. Nevertheless, a lot remains unknown about the clinical significance of oxidized albumin in other conditions.

In this study, we aimed to investigate the relationship between oxidized albumin levels in critically ill Covid-19 and hospital mortality as primary outcome measure. Secondary outcome measures were 30-day and ICU mortality, ICU length of stay and duration of invasive ventilation. Additionally, we aimed to compare the levels of oxidized albumin between critically ill Covid-19 patients and healthy controls. We hypothesized that critically ill Covid-19 patients had higher levels of oxidized albumin compared to healthy controls, and higher levels of oxidized albumin were associated with higher hospital mortality.

## Materials and methods

This single-center, retrospective cohort study was conducted in the ICUs of Karolinska University Hospital, Stockholm, Sweden.

### Study population

Adult (≥ 18 years) patients admitted to the ICU due to a Covid-19 infection between April 6, 2020, and May 16, 2021, were prospectively included in a biobank. The diagnosis of Covid-19 was based on either a positive polymerase chain reaction (PCR) test on a nasopharyngeal swab or on clinical judgement (International Classification of Disease codes U07.1 and U07.2), only patients with a positive PCR were included in the analyses.

Patients were excluded from the analyses if no sample to measure oxidized albumin was collected within the first 48 h of admission.

Biobank samples from previously recruited healthy volunteers were used to determine the normal range of oxidized albumin [18].

### Data collection

Outcome measures were retrospectively evaluated from the electronic patient records. Mortality was evaluated at hospital discharge and 30 days after the start of ICU treatment and at ICU discharge. Secondary outcome measures were assessed using the admission and discharge date and the registered ventilation modes.

All comorbidities, clinical parameters laboratory values and administered treatment were retrospectively extracted from the electronic patient records. Comorbidities were defined using the Charlson Comorbidity Index.

SOFA scores were calculated from the clinical data for the moment of admission and every 24 hours afterward. The highest SOFA score during the ICU admission (Maximum SOFA), the SOFA score at admission (Admission SOFA) and the SOFA score after 24 hours on the ICU (SOFA on the first ICU-day) were used for the analyses.

### Sample collection

Blood samples were collected and included in the biobank for all Covid-19 patients treated in the ICU within 48 hours after admission.

Blood samples were collected in EDTA tubes (PPT—BD biosciences, 362788).

After collection the samples were centrifuged at 4 °C, 2000*g*, and stored at − 80 °C in a biobank until analysis.

### Laboratory methods

To determine the oxidation state of Cys-34 in albumin, high performance liquid chromatography (HPLC) with fluorescence detection was used. We adapted previously developed protocols for the measurement of oxidized albumin [2, 19].

The HPLC protocol is presented in the supplement.

### Statistical analysis

Continuous variables are expressed as mean ± standard deviation (SD) for normally distributed data, or as median [minimum, maximum] for non-normally distributed data. Categorical variables are presented as count and percentage of the group (%).

Statistical comparison for continuous variables was performed by T-tests for groups with normal distribution or a sample size over 30, and Mann–Whitney U test for groups with a skewed distribution and a small sample size [20, 21]. Categorical variables were analyzed using Chi-square or Fisher’s exact test as appropriate.

The comparison of oxidized albumin levels was performed with Mann–Whitney U test for all three isoforms.

The clinical relevance of (elevated levels of) oxidized albumin was assessed using descriptive statistics. The study cohort was divided in three tertiles based on HNA-1 levels, after which the highest and lowest tertile were compared. For HNA-2, the cohort was dichotomized based on the presence of HNA-2. Comparisons for both HNA-1 and HNA-2 were conducted using the statistical methods described above. Due to lower-than-expected mortality rates and HNA-2 levels, a multivariable analysis could not be reliably performed.

A post hoc analysis was performed to examine the correlation between the highest creatinine value in the first 24 h after admission and the oxidized albumin fraction. This association was evaluated using a multivariable linear model, adjusting for age and the presence of chronic kidney disease before ICU admission.

Pairwise deletion was performed for missing values.

A two-sided *p*-value < 0.05 was considered significant.

All analyses were performed using the R Statistical language (version 4.3.2; R Core Team, 2023).

### Ethical considerations

Ethical approval was granted by the Swedish Ethical Review Authority (approval 2020-01302 and 2012/753-31/2). Informed consent was obtained from the patients and control group before blood samples were drawn or retrospectively (within 90 days but before any analysis was performed) when patients were unable to consent at inclusion. Participation in this study was voluntary, and consent could be revoked at any moment.

## Results

### Baseline characteristics

In total, 205 patients collected in the biobank were assessed for eligibility in this study. After excluding 19 (9.3%) patients without blood samples within 48 h after admission and 22 (10.7%) patients without positive PCR tests, 164 patients remained for the analyses.

Most of the patients were male (76.8%, 126/164) with a mean age of 60.3 years. The median length of ICU stay was 7.5 days (0.3–60.4). Out of the 164 patients, 31 died in the ICU (18.9%). Seventy-six patients (46.3%) required invasive ventilation, with a median duration of 11.4 days (0.2–53.0). The mean BMI was 29.3 (± 6.1) kg/m^2^, and the most common comorbidities were diabetes mellitus (31.1%, 51/164), chronic obstructive pulmonary disease (18.3%, 30/164) and kidney disease (8.5%, 14/164). Of the 164 patients, 49 (29.9%) of the patients were admitted after December 27 2020, the start date of the Covid-19 vaccination campaign in Sweden. The vaccination status of the majority (30/49, 61.2%) is unknown, 19 (38.8%) patients were not vaccinated. Baseline characteristics and treatment of the patients are presented in Table 1 and the supplement (Tables S1 and S2), baseline characteristics for the healthy volunteers are described in Table S3.

**Table 1:** Baseline characteristics

	Overall (*N* = 164)
Age (years)	60.3 (11.9)
Male sex—*n* (%)	126 (76.8%)
Mortality—*n* (%)	
Hospital mortality	29 (17.7%)
30-day mortality	31 (18.9%)
ICU^a^ mortality	25 (15.2%)
Length of ICU^a^ stay (days)	7.5 [0.3, 60.4]
Invasive ventilation	76 (46.3%)
BMI^b^ (kg/m^2^)	29.3 (6.1)
Scores	
Charlson comorbidity index	1.0 [0, 8.0]
Admission SOFA^c^ score	3.0 [0, 12.0]
SOFA^c^ score on first ICU^a^ Day	5.0 [2.0, 18.0]
Maximum SOFA^c^ score	6.0 [2.0, 19.0]
SAPS^d^ 3 score	54.0 (10.2)
Comorbidities—*n* (%)	
Diabetes mellitus	51 (31.1%)
Chronic obstructive pulmonary disease	30 (18.3%)
Chronic kidney disease	14 (8.5%)
Prior myocardial infarction	11 (6.7%)
Congestive heart failure	12 (7.3%)
Cerebrovascular disease	12 (7.3%)
Peripheral vascular disease	7 (4.3%)
Liver disease	6 (3.7%)

a: Intensive care unit, b: body mass index, c: Sequential Organ Failure Assessment, d: Simplified Acute Physiology Score. Presented as mean (SD) or median [minimum, maximum]

### Comparison of albumin fractions

Both HMA and HNA-1 were present in all Covid-19 patients, whereas HNA-2 was present in only 18% of the patients (*n* = 33). HNA-1 fractions were higher in Covid-19 patients (*n* = 164) than in healthy controls (*n* = 10). The mean HNA-1 fraction was 5.1 percent point higher (*p* = 0.010), whereas there was no significant difference in HNA- 2 between the two groups. HMA fractions were 6.2 percent point (*p* < 0.01) lower in the Covid-19 group.

Post hoc power calculations showed a power > 80% for all three comparisons between the study group and healthy controls.

**Fig. 1:**
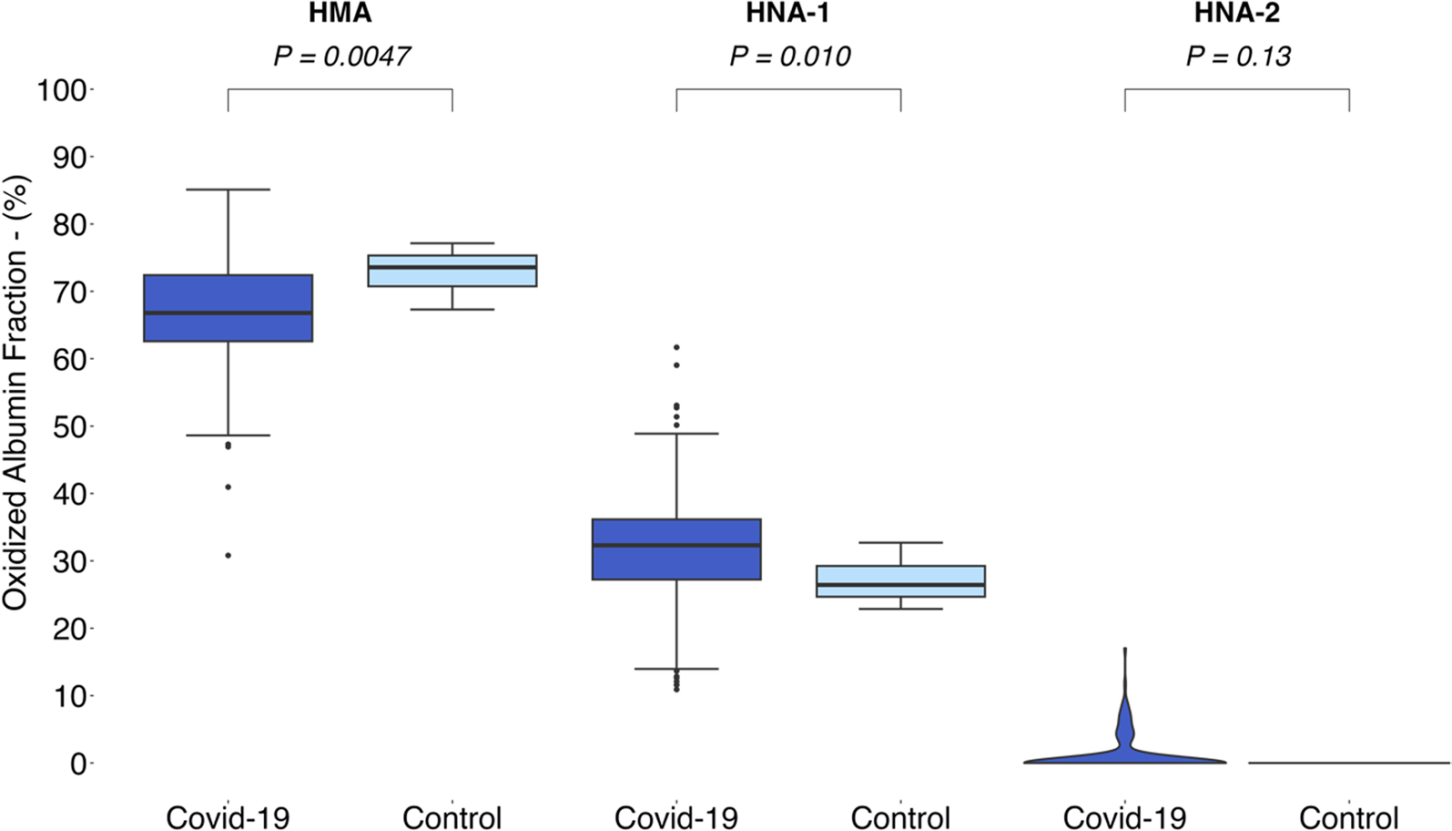
Oxidized albumin fractions in Covid-19 patients and healthy controls. The horizontal line within each box represents the median, the lower and upper borders of each box represent the first and third quartiles, respectively. T bars represent the differences between the lower and upper borders multiplied by 1.5. Outliers are plotted as single points. P-values by Mann–Whitney U test

A summary of the results is presented in Fig. 1 as well as Table S4 in the supplements. Table S4 also shows the different statistical analyses which were used for comparison between the groups.

#### Comparison between highest and lowest tertile based on HNA-1 fractions

A comparison of the tertiles with the highest and the lowest HNA-1 levels revealed no difference in any of the determined outcome variables. Mortality rates, admission duration, number of patients requiring invasive ventilation and duration of invasive ventilation were not significantly different between the two tertiles.

Patients with higher HNA-1 levels had higher SOFA scores compared to patients with low levels of HNA-1. Admission SOFA, SOFA on the first ICU Day and maximum SOFA score were all significantly higher in the highest tertile. The largest difference was found in the SOFA score on the first ICU Day, which was 2.0 points higher (95% CI 1.1–3.0, *p* < 0.001) in the high HNA-1 group compared to the low HNA-1 group.

Furthermore, the tertile with the highest HNA-1 levels, patients were 6.2 years (95% CI 1.7–10.6, *p* = 0.007) older and had higher maximum creatinine values in the first day of ICU admission compared to patients in the tertile with the low HNA-1 levels. The mean difference in creatinine values was 47.8 µmol/L (95% CI 8.8–86.5, *p* = 0.016). A sensitivity analysis excluding patients receiving albumin supplementation showed no significant difference in mortality rates, admission duration or number of patients requiring invasive ventilation either.

An extensive overview of the results is presented in Table 2, the results of the sensitivity analysis are displayed in table S5.

**Table 2:** Comparison of highest and lowest tertile based on HNA-1^a^ fractions

	Lowest tertile (*N* = 55)	Highest tertile (*N* = 55)	*p*-value
Male sex—*n* (%)	42 (76.4%)	43 (78.2%)	1.0
**Age (years)**	**56.5 (12.6)**	**62.6 (10.8)**	**0.007**
Outcome—*n* (%)			
Hospital mortality	10 (18.2%)	11 (20.0%)	1.0
30-day mortality	11 (20.0%)	12 (21.8%)	1.0
ICU mortality	8 (14.5%)	10 (18.2%)	0.80
Duration (days)			
ICU admission	6.83 [0.585, 54.4]	7.92 [0.253, 39.7]	0.73
Invasive ventilation	11.7 [1.83, 50.3]	10.1 [0.233, 36.9]	0.91
Non-invasive ventilation	3.75 [0.726, 14.8]	2.01 [0.0417, 10.0]	0.11
High flow nasal cannula	1.51 [0.0208, 9.06]	1.70 [0.0347, 31.5]	0.34
CRRT	19.0 [10.0, 28.0]	10.1 [2.54, 27.6]	0.39
BMI^b^ (kg/m^2^)	29.1 (6.69)	29.1 (6.21)	0.99
Comorbidities—*n* (%)			
Diabetes mellitus	16 (29.1%)	19 (34.5%)	0.68
Chronic pulmonary disease	12 (21.8%)	6 (10.9%)	0.20
Chronic kidney disease	2 (3.6%)	9 (16.4%)	0.057
Prior myocardial infarction	3 (5.5%)	3 (5.5%)	1.0
Congestive heart failure	2 (3.6%)	5 (9.1%)	0.44
Cerebrovascular disease	1 (1.8%)	6 (10.9%)	0.11
Peripheral vascular disease	1 (1.8%)	2 (3.6%)	1.0
Liver disease	2 (3.6%)	3 (5.5%)	1.0
Scores			
Charlson score	1.00 [0, 8.00]	1.00 [0, 8.00]	0.15
**Admission SOFA**^**c**^ **score**	**3.00 [1.00, 8.00]**	**5.00 [1.00, 8.00]**	**0.001**
**SOFA**^**c**^** score on first ICU day**	**3.00 [2.00, 11.0]**	**6.00 [3.00, 18.0]**	** < 0.001**
**Maximum SOFA**^**c**^ **score**	**6.00 [2.00, 12.0]**	**8.00 [3.00, 19.0]**	**0.009**
**SAPS**^**d**^ **II score**	**51.5 (8.78)**	**55.5 (10.8)**	**0.037**
Supportive therapy in the ICU—*n* (%)			
Invasive mechanical ventilation	22 (40.0%)	31 (56.4%)	0.13
Non-invasive mechanical ventilation	17 (30.9%)	22 (40.0%)	0.43
High-flow nasal cannula	20 (36.4%)	15 (27.3%)	0.41
Prone positioning	21 (38.2%)	22 (40.0%)	1.0
ECMO	0 (0%)	1 (1.8%)	1.0
CRRT	2 (3.6%)	7 (12.7%)	0.16
Medication administered in the intensive care—*n* (%)			
LMWH^e^	31 (56.4%)	40 (72.7%)	0.11
Corticosteroids	27 (49.1%)	25 (45.5%)	0.85
IL-blocking therapy^f^	7 (12.7%)	4 (7.3%)	0.53
Remdesivir	3 (5.5%)	3 (5.5%)	1.0
Albumin supplementation †	0 (0%)	2 (3.6%)	0.50
Laboratory and clinical parameters*			
Lowest pH	7.45 [7.14, 7.52]	7.46 [6.98, 7.52]	0.63
**Highest creatinine (μmol/L)**	**70.1 (45.0)**	**118 (134)**	**0.018**
Highest bilirubin (μmol/L)	8.64 (5.82)	8.57 (5.45)	0.95
P/F-ratio	12.6 (8.05)	12.1 (8.30)	0.75
Highest heart rate (/min)	89.5 (21.1)	93.7 (17.2)	0.25
Lowest systolic blood pressure (mmHg)	122 (25.9)	132 (33.6)	0.10
Albumin level at admission (g/L)	24.5 (3.98)	24.9 (3.76)	0.66
Albumin fractions (%)			
**HMA**^**g**^**fraction**	**75.4 (4.77)**	**57.9 (6.98)**	**< 0.001**
**HNA-1**^**a**^ **fraction**	**22.5 (5.77)**	**41.5 (6.39)**	**< 0.001**
**HNA-2**^**a**^ **fraction**	**0 [0, 17.0]**	**0 [0, 11.7]**	**0.008**

a: Human non-mercaptalbumin 1/2, b: intensive care unit, c: continuous renal replacement therapy, d: body mass index, e: Sequential Organ Failure Assessment, f: Simplified Acute Physiology Score, g: extracorporeal membrane oxygenation, h: low molecular weight heparin, i: interleukin blocking therapy (e.g., tocilizumab), j: human mercaptalbumin. * Values from the first 24 h after ICU admission. ^†^ Before or on day of sample. Presented as mean (SD) or median [minimum, maximum]. Statistically significant differences are shown in bold

#### Comparison between patients with and without HNA-2

None of the determined outcome variables differed between the groups with and without HNA-2. The only statistically significant difference between the groups were the HNA-1 levels, which were significantly higher in the group where HNA-2 was absent, and the number of patients receiving High-flow nasal cannula oxygen support. Furthermore, no statistically significant differences were found between the two groups regarding baseline characteristics, treatment, clinical parameters or laboratory values. For HNA-2, a sensitivity analysis excluding patients receiving albumin supplementation before sampling showed no differences in mortality rates, admission duration or number of patients requiring invasive ventilation either.

An extensive overview of the results is presented in Table 3, the results of the sensitivity analysis are presented in Table S6.

**Table 3:** Comparison between groups with and without HNA-2^a^

	HNA-2^a^ present (*N* = 33)	HNA-2^a^ absent (*N* = 153)	*p*-value
Male sex—*n* (%)	25 (80.6%)	101 (75.9%)	0.75
Age (years)	61.0 (12.4)	60.2 (11.8)	0.73
Outcome—*n* (%)			
ICU mortality	4 (12.9%)	21 (15.8%)	1.0
Hospital mortality	5 (16.1%)	24 (18.0%)	1.0
180-day mortality	6 (19.4%)	26 (19.5%)	1.0
Duration (days)			
Duration of admission	8.88 [0.969, 54.4]	6.80 [0.253, 60.4]	0.99
Invasive ventilation	9.07 [1.31, 50.3]	12.7 [0.233, 53.0]	0.76
Non-invasive ventilation	4.34 [0.115, 8.87]	3.50 [0.0417, 28.6]	0.99
High-flow nasal cannula	4.54 [0.0347, 9.06]	1.89 [0.0208, 31.5]	0.73
CRRT	10.1 [2.54, 28.0]	9.95 [3.09, 30.6]	0.91
BMI^b^ (kg/m^2^)	29.4 (8.28)	29.3 (5.40)	0.97
Comorbidities—*n* (%)			
Diabetes mellitus	6 (19.4%)	45 (33.8%)	0.18
Chronic pulmonary disease	4 (12.9%)	26 (19.5%)	0.55
Chronic kidney disease	2 (6.5%)	12 (9.0%)	1.0
Prior myocardial infarction	2 (6.5%)	9 (6.8%)	1.0
Congestive heart failure	3 (9.7%)	9 (6.8%)	0.70
Cerebrovascular disease	1 (3.2%)	11 (8.3%)	0.47
Peripheral vascular disease	2 (6.5%)	5 (3.8%)	0.62
Liver disease	1 (3.2%)	5 (3.8%)	1.0
Scores			
Charlson score	0 [0, 8.00]	1.00 [0, 8.00]	0.33
Admission SOFA^c^ score	4.00 [1.00, 12.0]	3.00 [0, 8.00]	0.12
SOFA^c^ score on first day in the intensive care unit	4.00 [2.00, 18.0]	5.00 [2.00, 12.0]	0.82
Maximum SOFA^c^ score	6.00 [3.00, 19.0]	6.00 [2.00, 19.0]	0.60
SAPS^d^ II score	52.0 [42.0, 108]	53.0 [32.0, 88.0]	0.17
Supportive therapy in the ICU—*n* (%)			
Invasive mechanical ventilation	14 (45.2%)	62 (46.6%)	1.0
Non-invasive mechanical ventilation	10 (32.3%)	45 (33.8%)	1.0
**High-flow nasal cannula**	**6 (19.4%)**	**54 (40.6%)**	**0.045**
Prone positioning	12 (38.7%)	51 (38.3%)	1.0
ECMO	0 (0%)	1 (0.8%)	1.0
Continuous renal replacement therapy	4 (12.9%)	10 (7.5%)	0.31
Medication administered in the intensive care—*n* (%)			
LMWH^e^	20 (64.5%)	87 (65.4%)	1.0
Corticosteroids	13 (41.9%)	74 (55.6%)	0.24
IL-blocking therapy^f^	2 (6.5%)	15 (11.3%)	0.74
Remdesivir	2 (6.5%)	8 (6.0%)	1.0
Albumin supplementation †	2 (6.5%)	3 (2.3%)	0.24
Laboratory and clinical parameters*			
Lowest pH	7.46 [6.98, 7.50]	7.46 [7.14, 7.53]	0.46
Highest creatinine (μmol/L)	85.7 (54.5)	90.0 (92.2)	0.81
Highest bilirubin (μmol/L)	10.1 (7.61)	8.79 (5.07)	0.27
P/F-ratio	12.4 (6.62)	12.5 (10.1)	0.95
Highest heart rate (/min)	91.8 (19.0)	91.7 (18.9)	0.97
Lowest systolic blood pressure (mmHg)	127 (29.4)	126 (28.9)	0.90
Albumin level at admission (g/L)	24.2 (3.48)	24.9 (3.66)	0.35
Albumin fractions			
HMA^**g**^ fraction	70.2 (12.4)	66.0 (7.52)	0.074
**HNA-1**^**a**^** fraction**	**23.9 (11.6)**	**34.0 (7.52)**	**< 0.001**
**HNA-2**^**a**^ **fraction**	**5.11 [1.80, 17.0]**	**0 [0, 0]**	**< 0.001**

a: Human non-mercaptalbumin 1/2, b: intensive care unit, c: continuous renal replacement therapy, d: body mass index, e: Sequential Organ Failure Assessment, f: Simplified Acute Physiology Score, g: extracorporeal membrane oxygenation, h: low molecular weight heparin, i: Interleukin blocking therapy (e.g., tocilizumab), j: human mercaptalbumin. * Values from the first 24 h after ICU admission. ^†^ Before or on day of sample. Presented as mean (SD) or median [minimum, maximum]. Statistically significant differences are shown in bold

#### Correlation between oxidized albumin and creatinine

The association between the oxidized albumin fraction (sum of HNA-1 and HNA-2) and maximum creatinine value in the first 24 hours of admission was further examined with a linear regression model. Logarithmic transformation was performed on creatinine values to approach a normal distribution.

After excluding patients with missing data for both kidney disease and maximum creatinine values, the data of 154 patients were used for the analysis.

**Fig. 2:**
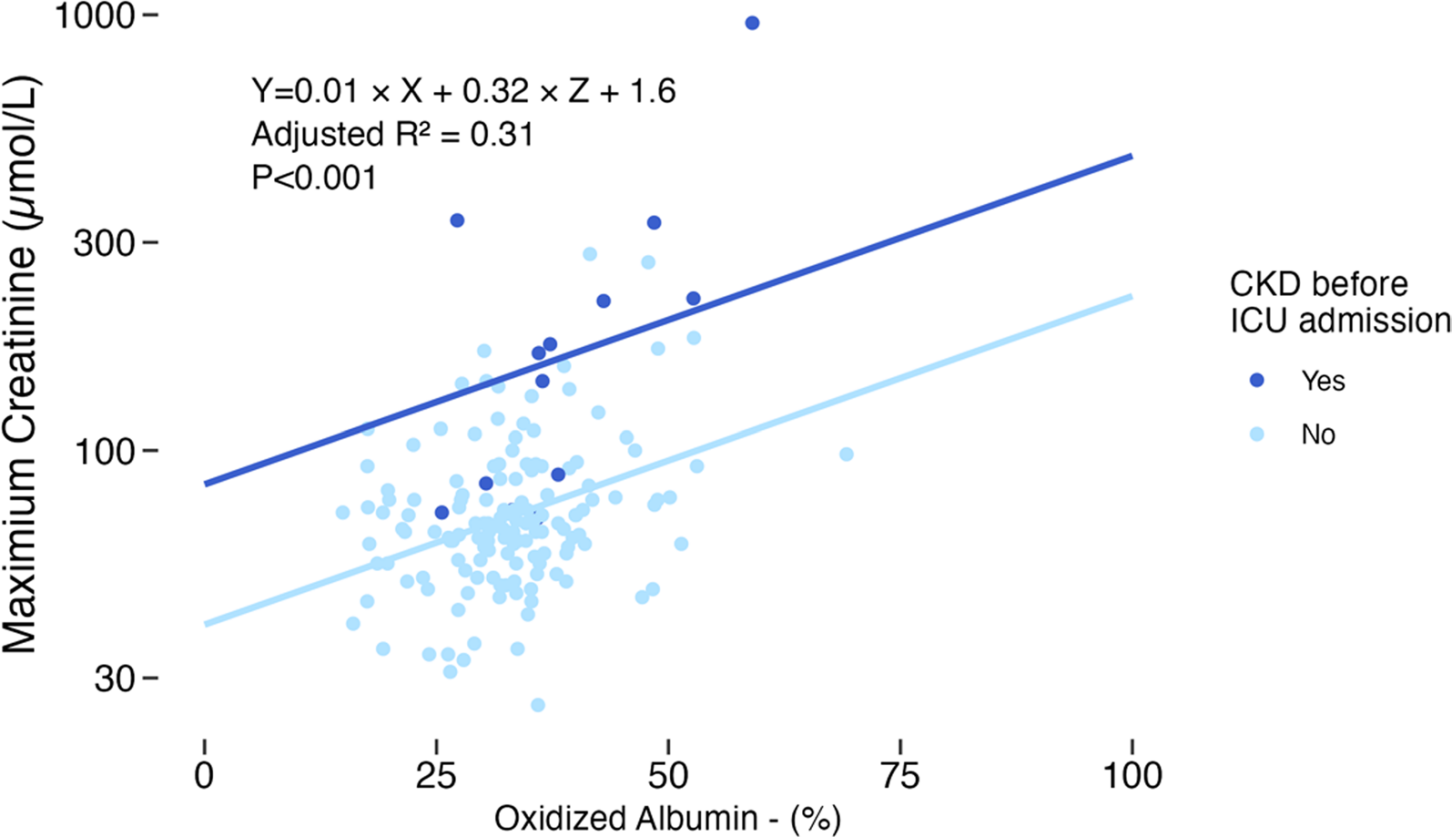
Correlation plot with oxidized albumin fractions and creatinine levels, regression lines for both patients with and without prior chronic kidney disease, corrected for age. CKD: chronic kidney disease, ICU: intensive care unit

The linear regression showed that the fraction of oxidized albumin correlates weakly to the highest creatinine levels in the first 24 h of admission, independent of age or the presence of chronic kidney disease before admission to the ICU (Table 4 and Fig. 2).

**Table 4:** Multivariable linear regression coefficients

Predictors for high creatinine	*B*	95%-CI	*p*-value
Oxidized albumin fraction	0.01	0.00–0.01	< 0.001
CKD before ICU admission [Yes = 1]	0.32	0.22–0.43	< 0.001
Adjusted *R*^2^: 0.31			

Corrected for Age

*CKD* chronic kidney disease, *ICU* intensive care unit

## Discussion

To our knowledge, this is the first study to assess levels of oxidized albumin in critically ill Covid-19 patients, and evaluate the association between oxidized albumin and mortality, length of stay and duration of invasive ventilation. As hypothesized, Covid-19 patients had higher levels of single oxidized albumin. However, no differences were found in HNA-2 or the outcome measures in patients in whom HNA-1 levels were higher or HNA-2 was present, compared to patients in whom HNA-1 levels were lower or HNA-2 was absent. These findings suggest that oxidized albumin is not a risk factor for, or an accurate predictor of, mortality, length of stay or duration of invasive ventilation in critically ill Covid-19 patients. However, oxidized albumin seems to be related to organ failure. Patients with higher HNA-1 levels had higher SOFA scores and maximum creatinine value in the first 24 hours of admission. These creatinine values correlated weakly with the total fraction of oxidized albumin, the sum of HNA-1 and HNA-2, in a post hoc linear model.

Even though a difference in fractions of oxidized albumin between Covid-19 patients and healthy controls was observed, the difference is not as large as one might expect based on previous research in other diseases [2, 8]. No studies describing oxidized albumin levels in Covid-19 patients are present till this day, however, studies on sepsis and liver failure show higher levels of both HNA-1 and HNA-2 in both diseases [2, 8].

Elevated levels of oxidized albumin are the result of either increased formation or decreased degradation. Even though the general amount of oxidative stress and antioxidant capacity were not measured, we believe that the slight elevation of oxidized albumin is the result of oxidative stress that is associated with the critical illness. There are studies that suggest a role of oxidative stress in the pathophysiology of Covid-19, which, in turn, could lead to increased formation of oxidized albumin [10, 22]. Regarding metabolism, one recent study showed evidence that the liver is the main site for the breakdown of oxidized albumin [23]. The study cohort contained a very low number of patients with history of liver disease or patients with indications for (severe) acute liver failure. Therefore, patients should be capable of metabolizing oxidized albumin and remaining relatively normal levels of oxidized albumin. Summarized, the increase of oxidized albumin may be driven by higher levels of oxidative stress but compensated by (hepatic) metabolism. This could explain the fact that levels of oxidized albumin are only minimally increased in Covid-19 patients compared to healthy controls.

In this study, there was no association between oxidized albumin levels and ICU mortality, length of stay or duration of mechanical ventilation. Interestingly, a previous study in liver failure patients showed an association between HNA-2 levels and short-term mortality. The optimal cut-off point for mortality prediction in this study was at an HNA-2 fraction of 12% [2]. HNA-2 is present in a small portion of the patients (circa 18%). When HNA-2 is present, the levels are relatively low. Only one patient had an HNA-2 fraction higher than 12%. Because of the low number of patients with HNA-2, it is hard to perform a proper analysis and find a cut-off point for HNA-2 that is appropriate for predicting mortality in this cohort. Besides, there is no significant difference in HNA-2 levels between the cohort and control group, this could also explain the absence of an association with mortality. For HNA-1, previous studies in hemodialysis patients showed a small increase in long term mortality with higher levels of HNA-1, however this association was not as strong as for HNA-2 in liver failure [16]. The results of this study suggest that neither HNA-1 nor HNA-2 are associated with higher ICU mortality, longer length of stay or longer duration of mechanical ventilation and therefore no safe HNA-1 or HNA-2 level, which predicts survival rates, exists.

On the other hand, when looking at SOFA scores, a difference in mortality might be expected between patients with high and low levels of HNA-1. Higher SOFA scores are associated with higher mortality rates [24]. It is possible that the study was underpowered to show a difference in mortality between patients with high and low levels HNA-1, since there is a small, but not statistically significant difference in mortality. With the small differences in mortality found between the two groups, the correct sample size would be little under 7500 patients per group for HNA-1. The SOFA scores of the HNA-1 tertiles may be explained by the difference in maximum creatinine values; creatinine and urine output are the two factors that determine the SOFA score. In the tertile with high HNA-1 levels, the maximum creatinine values within the first 24 h of admission were higher than in the tertile with low HNA-1 levels, which in turn may increase the SOFA score.

The linear regression model shows a weak positive correlation between the fraction of oxidized albumin and creatinine levels. Prior studies regarding oxidized albumin in renal failure were focused on patients with chronic kidney disease, but this study shows that renal function in general could be correlated to the fraction of oxidized albumin [6, 15, 17, 25, 26]. One previous study also assessed a correlation between the total fraction of oxidized albumin and renal function, when looking at the glomerular filtration rate (GFR) [26]. The GFR is inversely correlated to creatinine levels, which are used in the calculation of the GFR [27]. In this study, higher fractions of oxidized albumin correlated with higher creatinine levels, which is in line with the findings in a previous study, where higher levels of oxidized albumin correlate to lower glomerular filtration rates [26]. Some studies on oxidized albumin in chronic kidney disease suggest that oxidized albumin is a biomarker for oxidative stress, while others suggest that oxidized albumin is also involved in the development of several renal diseases such as focal glomerulosclerosis and elevated levels of oxidized albumin may also contribute to the progression, rather than merely being a biomarker [4, 28]. The results of this study cannot be used to support either of these theories. However, the association between oxidized albumin and creatinine or GFR may also imply that the kidney plays a role in the elimination of oxidized albumin. The kidney plays a minor role in the elimination of non-oxidized albumin, but little is known about the metabolism and elimination of oxidized albumin [29]. Increasing rates of oxidized albumin with decreasing glomerular filtration rates may therefore imply that the former is caused by the latter.

Commercial albumin solutions used for infusion contain relatively high levels of HNA-1 and HNA-2 compared to albumin fractions in plasma, which was also seen in our preliminary results (Electronic supplement Figure S1) [30]. Therefore, albumin supplementation could influence the levels of oxidized albumin in patients. As HNA-2 was only present in a few patients and at low levels, one of our concerns after the initial analyses was that the presence of HNA-2 was only determined by supplementation with albumin. However, only 9% of the patients in whom HNA-2 was present, received albumin supplementation before the moment of sampling. This is by no means direct evidence that albumin supplementation does not influence oxidized albumin fractions at all, but since 91% of the patients with HNA-2 did not receive albumin supplementation, the presence of HNA-2 is not merely the result of albumin supplementation. This is supported by the absence of major differences in the sensitivity analyses excluding patients who received albumin supplementation. Other studies have shown that albumin supplementation only has a small effect on albumin levels shortly after supplementation but does not affect oxidized albumin levels on later timepoints [30]. The presence of HNA-2 in the patients who did not receive supplementation may therefore be a result of oxidative stress, or some other pathophysiological process that we do not understand yet.

In the current ICU practice, where Covid-19 cases are less frequent, oxidized albumin may still be of interest. Contrary to the findings in patients with Covid-19 in this study, higher levels of oxidized albumin, were associated to higher mortality in patients with sepsis and liver failure [2, 31]. This association was observed for the HNA-2 isoform in both diseases. Future research may be directed to patients with high amounts of the HNA-2 isoform and have high levels of oxidative stress. In these populations, HNA-2 may be used to quantify oxidative stress, disease severity or even predict mortality.

### Strengths and limitations

This study was a retrospective study; therefore, the ability to perform additional laboratory analyses and measurements was limited. Secondly, during the Covid-19 pandemic, new ICU beds were established to deal with the extremely large numbers of patients. In these wards charting was done on paper charts instead of electronic patient records. These data have been digitalized, but we cannot be sure that the data are as complete as they would have been when the charting would have been done in an electronic patient record right away. However, all data on the primary and secondary outcome measures were complete. Therefore, the results of the primary analyses are valid and reliable. Overall, there was only a small amount of missing data in a large cohort, except for the variable BMI.

For the comparison of albumin fractions, there is a relatively small control group. Besides, the control group was younger than the cohort, and since aging has been shown to possibly increase oxidized albumin, the actual difference between the groups might be smaller [26].

Even though the sample size of this study was relatively large, it may have been too small to detect a significant difference in mortality between the groups; since the number of patients in which HNA-2 was detected was low and the mortality rates for Covid-19 were low compared to the mortality rates published in international studies.

## Conclusion

In conclusion, the results from this research suggest that the fractions of HNA-1 are higher in critically ill Covid-19 patients than in healthy controls. Higher levels of oxidized albumin are not associated with higher 30-day, hospital or ICU mortality, longer length of stay or longer duration of invasive ventilation. Patients with higher levels of oxidized albumin showed higher levels of creatinine and SOFA scores.

Even though this research did not provide new prognostic tools for mortality and admission duration, it contributed to the knowledge about oxidized albumin in critically ill Covid-19 patients and has provided new insights which can be used to direct new studies.

## Supplementary Information

Supplementary Material 1.

## Data Availability

The datasets used and/or analyzed during the current study are available from the corresponding author on reasonable request.

## Abbreviations

/L: Per liter
BMI: Body mass index
CI: Confidence interval
CRRT: Continuous renal replacement therapy
Cys-34: Cysteine 34
ECMO: Extra corporeal membrane oxygenation
EDTA: Ethylenediaminetetraacetic acid
GFR: Glomerular filtration rate
HMA: Human mercaptalbumin
HNA-(1/2): Human non-mercaptalbumin (1/2)
HPLC: High performance liquid chromatography
ICU: Intensive care unit
IL: Interleukin
LMWH: Low molecular weight heparin
MELD: Model of end stage liver disease
mmol: Millimole
P/F Ratio: Arterial partial pressure of oxygen/fraction of inspired oxygen ratio
PaO2: Arterial partial pressure of oxygen
PCR: Polymerase chain reaction
PPT: Plasma preparation tube
SAPS: Simplified Acute Physiology Score
SD: Standard deviation
SOFA: Sequential Organ Failure Assessment
µmol: Micromole
