# Supplementary material for: Oxidized albumin and its association with mortality in critically ill Covid-19 patients: a retrospective cohort study
Source: Intensive Care Med Exp. 2026 Mar 2;14:25. doi: 10.1186/s40635-026-00872-x (PMC12953812; doi:10.1186/s40635-026-00872-x)
Supplement: Supplementary file 1 — Supplementary Material 1. [file 40635_2026_872_MOESM1_ESM.docx]

**SUPPLEMENT TO:**

**Oxidized albumin and its association with mortality in critically ill Covid-19 patients: a retrospective, single-center cohort study.**

Teun Aben ^1,2^, Johan Helleberg ^1,2^, Jonathan Grip ^1,2^, Olav Rooijackers ^1, 2^.

1: Department of Clinical Science Intervention and Technology (CLINTEC), Division of Anaesthesiology and Intensive Care, Karolinska Institutet, Huddinge, Sweden.

2: Department of Perioperative Medicine and Intensive Care, Karolinska University Hospital, Huddinge, Sweden.

**High Performance Liquid Chromatography Protocol:**

At first, plasma samples were diluted to a ratio of 3:100 with a phosphate buffer containing 0.1 M sodium phosphate and 0.3 M sodium chloride, pH 6.87. Then, the sample was filtered through a 0.45 µm filter.

After filtration, 20 µL of the solution was injected into the HPLC system (Waters Arc). Albumin fractions were separated using an anion exchange column (Shodex ASAHIpak ES-502 N 7C, 7.5 mm x 100 mm), with a 50 mM sodium acetate, 400 mM sodium sulphate, pH 4.85 buffer as a mobile phase. Ethanol was used for elution (gradient 0 to 6%, flow 1 millilitre per minute). The temperature in the column was kept constant at 35 degrees Celsius.

Fluorescence detection was carried out at wavelengths between 280 and 340 nm by a Waters 2474 FLR detector. This resulted in a chromatogram with three peaks.

Quantification of the peaks was performed by Empower 3.0 (Waters). Oxidized albumin fractions were calculated by dividing the area under the curve (AUC) of the desired peak by the sum of the AUC for all the chromatogram peaks for albumin (for example: Fraction of HMA (%) = AUC_HMA_/ (AUC_HMA_ + AUC_HNA-1_ +AUC_HNA-2_) x 100%).

A commercial albumin solution with 20% albumin was used as a quality control.

Table S1: Treatment received in the ICU

| ***Table S1: Treatment received in the ICU ᵃ*** | |
| --- | --- |
|  | **Overall (N=164)** |
| **Medication administered in the ICU ᵃ - n (%)** | |
| LMWH ^b^ | 107 (65.2%) |
| Corticosteroids | 87 (53.0%) |
| Albumin | 38 (23.2%) |
| IL-blocking therapy ^c^ | 17 (10.4%) |
| Remdesivir | 10 (6.1%) |
| **Supportive Therapy in the ICU ᵃ - n (%)** | |
| Non-Invasive Ventilation | 55 (33.5%) |
| High Flow Nasal Cannula | 60 (36.6%) |
| Prone Positioning | 63 (38.4%) |
| ECMO ^d^ | 1 (0.6%) |
| CRRT ^e^ | 14 (8.5%) |
| **Duration (Days)** | |
| Invasive Ventilation | 11.4 [0.2, 53.0] |
| Non-Invasive Ventilation | 3.9 [0.04, 28.6] |
| Optiflow | 2.1 [0.02, 31.5] |
| CRRT ^e^ | 10.0 [2.5, 30.6] |
| a: Intensive Care Unit, b: Low Molecular Weight Heparin, c: Interleukin Blocking therapy (eg. Tocilizumab), d: Extra Corporeal Membrane Oxygenation, e: Continuous Renal Replacement Therapy Presented as Mean (SD) or Median [Minimum, Maximum] | |

Table S2: Laboratory and clinical parameters within 24 hours after admission.

|  | |
| --- | --- |
|  |  |
|  |  |
|  |  |
|  |  |
|  |  |
|  |  |
|  |  |
|  |  |
|  |  |
|  |  |
|  |  |
|  |  |
|  |  |
|  |  |
|  |  |
|  |  |
|  |  |
|  |  |
|  | |

| ***Table S2: Laboratory and clinical parameters within 24 hours after admission*** | |
| --- | --- |
|  | **Overall (N=164)** |
| **Clinical Parameters** |  |
| Highest Temperature (°C) | 37.6 (0.9) |
| Lowest Systolic Blood Pressure (mmHg) | 126 (28.9) |
| Highest Heart Rate (/min) | 91.7 (18.9) |
| Glasgow Coma Scale | 15.0 [3.0, 15.0] |
| **Laboratory Parameters** |  |
| Lowest pH | 7.46 [6.98, 7.53] |
| PaO2 ᵃ (kPa) | 10.0 (9.7) |
| P/F-Ratio ᵇ (kPa) | 12.5 (9.5) |
| Highest Leukocyte count (×10⁹/L) | 9.5 (7.5) |
| Highest Creatinine (μmol/L) | 89.2 (86.3) |
| Highest Bilirubin (μmol/L) | 9.0 (5.6) |
| Albumin level at admission (g/L) | 24.7 (3.6) |
| **Albumin Fractions (%)** |  |
| HMA ^c^ fraction | 66.8 (8.8) |
| HNA-1 ᵈ fraction | 32.1 (9.3) |
| HNA-2 ᵈ fraction | 0 [0, 17.0] |
| a: Arterial partial pressure of oxygen, b: PaO2/FiO2, c: Human Mercaptalbumin, d: Human Non-Mercaptalbumin 1 and 2. Presented as Mean (SD) or Median [Minimum, Maximum] | |

Table S3: Control group characteristics

| ***Table S3: Control group characteristics*** | |
| --- | --- |
| Male Sex | 30% |
| Age | 45 [22-50] |
| BMI | 33 [29-33] |
| Displayed as Median [Minimum; Maximum] | |

| ***Table S4: Oxidized albumin fractions in Covid-19 & Healthy Controls.*** | | | | | |
| --- | --- | --- | --- | --- | --- |
|  | **Covid-19** |  | **Controls** | | |
|  | *Mean (%)* |  | *Mean (%)* | *Mean Difference* | *Statistical test* |
| **HMA** | 66.91 |  | 72.96 | 6.04 ** | Mann-Whitney U test |
| **HNA-1** | 32.04 |  | 27.04 | -5.00 * | Mann- Whitney U test |
| **HNA-2** | 1.05 |  | 0.00 | -1.05 | Mann-Whitney U test |
| Mean difference was calculated by subtracting the mean of the Covid-19 group from the mean of control group. * = P <0.05, ** = P<0.01. | | | | | |

Table S4: Oxidized albumin fractions in Covid-19 & Healthy Controls.

| **Table S5: Sensitivity Analysis for HNA-1 tertiles** | | | |
| --- | --- | --- | --- |
|  | **Lowest Tertile (N=53)** | **Highest Tertile (N=53)** | **P-value** |
| **Male Sex - n (%)** | 41 (77.4%) | 41 (77.4%) | 1.0 |
| **Age (Years)** | **57.1 (12.1)** | **62.3 (10.9)** | **0.022** |
| **Outcome - n (%)** |  |  |  |
| Hospital Mortality | 10 (18.9%) | 10 (18.9%) | 1.0 |
| 30-day Mortality | 11 (20.8%) | 11 (20.8%) | 1.0 |
| ICU Mortality | 8 (15.1%) | 9 (17.0%) | 1.0 |
| **Duration (Days)** |  |  |  |
| ICU Admission | 6.83 [0.59, 54.4] | 7.43 [0.25, 39.7] | 0.86 |
| Invasive Ventilation | 11.7 [1.8, 50.3] | 10.1 [0.23, 36.9] | 0.99 |
| Non-Invasive Ventilation | 3.75 [0.73, 14.8] | 2.01 [0.042, 10.0] | 0.11 |
| High Flow Nasal Cannula | 1.47 [0.021, 9.1] | 2.00 [0.035, 31.5] | 0.30 |
| CRRT | 19.0 [10.0, 28.0] | 10.1 [2.5, 27.6] | 0.39 |
| **BMIᵇ (kg/m²)** | 28.7 (6.5) | 29.3 (6.3) | 0.73 |
| **Comorbidities - n (%)** |  |  |  |
| Diabetes Mellitus | 16 (30.2%) | 19 (35.8%) | 0.68 |
| Chronic Pulmonary Disease | 11 (20.8%) | 6 (11.3%) | 0.29 |
| Chronic Kidney Disease | 2 (3.8%) | 8 (15.1%) | 0.097 |
| Prior Myocardial Infarction | 3 (5.7%) | 2 (3.8%) | 1.00 |
| Congestive Heart Failure | 2 (3.8%) | 5 (9.4%) | 0.44 |
| Cerebrovascular Disease | 1 (1.9%) | 6 (11.3%) | 0.11 |
| Peripheral Vascular Disease | 1 (1.9%) | 2 (3.8%) | 1.00 |
| Liver Disease | 2 (3.8%) | 3 (5.7%) | 1.00 |
| **Scores** |  |  |  |
| Charlson Score | 1.0 [0, 8.0] | 1.0 [0, 8.0] | 0.24 |
| **Admission SOFAᶜ Score** | **3.0 [1.0, 8.0]** | **5.0 [1.0, 8.0]** | **0.0027** |
| **SOFAᶜ Score on first ICU day** | **3.0 [2.0, 11.0]** | **6.0 [3.0, 18.0]** | **<0.001** |
| **Maximum SOFAᶜ Score** | **6.0 [3.0, 12.0]** | **7.5 [3.0, 19.0]** | **0.026** |
| SAPSᵈ II Score | 52.0 (8.6) | 55.3 (10.8) | 0.084 |
| **Supportive Therapy in the ICU - n (%)** |  |  |  |
| Invasive Mechanical Ventilation | 22 (41.5%) | 29 (54.7%) | 0.24 |
| Non-Invasive Mechanical Ventilation | 17 (32.1%) | 21 (39.6%) | 0.54 |
| High Flow Nasal Cannula | 19 (35.8%) | 14 (26.4%) | 0.40 |
| Prone Positioning | 20 (37.7%) | 21 (39.6%) | 1.0 |
| ECMO | 0 (0%) | 1 (1.9%) | 1.0 |
| CRRT | 2 (3.8%) | 7 (13.2%) | 0.16 |
| **Medication administered in the Intensive Care - n (%)** | | | |
| **LMWHᵉ** | **29 (54.7%)** | **40 (75.5%)** | **0.042** |
| Corticosteroids | 27 (50.9%) | 24 (45.3%) | 0.70 |
| IL-blocking therapyᶠ | 7 (13.2%) | 4 (7.5%) | 0.52 |
| Remdesivir | 3 (5.7%) | 3 (5.7%) | 1.0 |
| **Laboratory and Clinical parameters*** |  |  |  |
| Lowest pH | 7.45 [7.14, 7.52] | 7.46 [6.98, 7.52] | 0.56 |
| **Highest Creatinine (μmol/L)** | **70.5 (45.8)** | **117 (135)** | **0.026** |
| Highest Bilirubin (μmol/L) | 8.7 (5.9) | 8.7 (5.5) | 0.99 |
| P/F-Ratio | 12.5 (8.2) | 12.3 (8.4) | 0.86 |
| Highest Heart Rate (/min) | 89.8 (21.2) | 93.7 (17.5) | 0.31 |
| Lowest Systolic Blood Pressure (mmHg) | 121 (26.0) | 131 (34.2) | 0.092 |
| Albumin level at admission (g/L) | 24.3 (3.96) | 25.2 (3.50) | 0.27 |
| **Albumin fractions (%)** |  |  |  |
| **HMAᵍ fraction** | **75.5 (4.8)** | **57.9 (6.9)** | **<0.001** |
| **HNA-1ᵃ fraction** | **22.3 (5.7)** | **41.6 (6.5)** | **<0.001** |
| **HNA-2ᵃ fraction** | **0 [0, 17.0]** | **0 [0, 11.7]** | **0.0024** |
| \| a: Human Non-Mercaptalbumin 1/2, b: Body Mass Index, c: Sequential Organ Failure Assesment, d: Simplified Acute Physiology Score, e: Interleukin Blocking therapy (eg. Tocilizumab), f: Low Molecular Weight Heparin, g: Human Mercaptalbumin. * Values from the first 24 hours after ICU admission. Normally distributed variables are presented as Mean (SD), variables with a skewed deviation as Median [Min, Max] \| \| --- \| | | | |

| **Table S6: Sensitivity Analysis for HNA-2** | | | |
| --- | --- | --- | --- |
|  | **HNA-2ᵃ Present (N=29)** | **HNA-2ᵃ Absent (N=130)** | **P-value** |
| **Male Sex - n (%)** | 23 (79.3%) | 99 (76.2%) | 0.90 |
| **Age (Years)** | 60.2 (12.5) | 59.9 (11.7) | 0.88 |
| **Outcome - n (%)** |  |  |  |
| ICU Mortality | 4 (13.8%) | 19 (14.6%) | 1.0 |
| Hospital Mortality | 5 (17.2%) | 22 (16.9%) | 0.99 |
| 180-day Mortality | 6 (20.7%) | 24 (18.5%) | 1.0 |
| **Duration (Days)** |  |  |  |
| Duration of admission | 8.0 [0.97, 54.4] | 6.7 [0.25, 60.4] | 0.99 |
| Invasive Ventilation | 10.6 [1.31, 50.3] | 13.2 [0.23, 53.0] | 0.88 |
| Non-Invasive Ventilation | 4.3 [0.12, 5.7] | 3.4 [0.042, 28.6] | 0.74 |
| High Flow Nasal Cannula | 3.4 [0.035, 9.1] | 2.0 [0.021, 31.5] | 1.0 |
| CRRT | 10.1 [2.5, 28.0] | 10.1 [3.1, 30.6] | 0.81 |
| **BMIᵇ (kg/m²)** | 29.4 (8.6) | 29.4 (5.4) | 0.99 |
| **Comorbidities - n (%)** |  |  |  |
| Diabetes Mellitus | 5 (17.2%) | 45 (34.6%) | 0.11 |
| Chronic Pulmonary Disease | 4 (13.8%) | 25 (19.2%) | 0.68 |
| Chronic Kidney Disease | 1 (3.4%) | 12 (9.2%) | 0.47 |
| Prior Myocardial Infarction | 2 (6.9%) | 8 (6.2%) | 1.0 |
| Congestive Heart Failure | 3 (10.3%) | 8 (6.2%) | 0.42 |
| Cerebrovascular Disease | 1 (3.4%) | 11 (8.5%) | 0.70 |
| Peripheral Vascular Disease | 1 (3.4%) | 5 (3.8%) | 1.0 |
| Liver Disease | 1 (3.4%) | 5 (3.8%) | 1.0 |
| **Scores** |  |  |  |
| Charlson Score | 0 [0, 8.0] | 1.0 [0, 8.0] | 0.17 |
| Admission SOFAᶜ Score | 3.5 [1.0, 12.0] | 3.0 [0, 8.0] | 0.48 |
| SOFAᶜ Score on first day in the Intensive Care Unit | 3.0 [2.0, 18.0] | 5.0 [2.0, 11.0] | 0.69 |
| Maximum SOFAᶜ Score | 6.0 [3.0, 19.0] | 6.0 [2.0, 19.0] | 0.59 |
| SAPSᵈ II Score | 51.0 [42.0, 108] | 53.0 [32.0, 88.0] | 0.24 |
| **Supportive Therapy in the ICU - n (%)** |  |  |  |
| Invasive Mechanical Ventilation | 13 (44.8%) | 59 (45.4%) | 1.0 |
| Non-Invasive Mechanical Ventilation | 8 (27.6%) | 44 (33.8%) | 0.67 |
| **High Flow Nasal Cannula** | **5 (17.2%)** | **52 (40.0%)** | **0.036** |
| Prone Positioning | 12 (41.4%) | 49 (37.7%) | 0.87 |
| ECMO | 0 (0%) | 1 (0.8%) | 1.0 |
| Continuous Renal Replacement Therapy | 4 (13.8%) | 9 (6.9%) | 0.26 |
| **Medication administered in the Intensive Care - n (%)** | | | |
| LMWHᵉ | 19 (65.5%) | 86 (66.2%) | 1.0 |
| Corticosteroids | 12 (41.4%) | 72 (55.4%) | 0.25 |
| IL-blocking therapyᶠ | 2 (6.9%) | 15 (11.5%) | 0.74 |
| Remdesivir | 2 (6.9%) | 8 (6.2%) | 1.0 |
| **Laboratory and Clinical parameters*** |  |  |  |
| Lowest pH | 7.46 [6.98, 7.50] | 7.46 [7.14, 7.53] | 0.42 |
| Highest Creatinine (μmol/L) | 78.0 (47.1) | 90.1 (92.8) | 0.51 |
| Highest Bilirubin (μmol/L) | 10.0 (7.5) | 8.8 (5.1) | 0.34 |
| P/F-Ratio | 12.5 (6.8) | 12.6 (10.2) | 0.94 |
| Highest Heart Rate (/min) | 91.0 (19.3) | 91.3 (18.8) | 0.93 |
| Lowest Systolic Blood Pressure (mmHg) | 127 (30.3) | 126 (28.8) | 0.83 |
| Albumin level at admission (g/L) | 24.6 (3.22) | 25.0 (3.60) | 0.57 |
| **Albumin fractions** |  |  |  |
| **HMAᵍ fraction** | **71.3 (11.9)** | **65.9 (7.6)** | **0.026** |
| **HNA-1ᵃ fraction** | **23.0 (11.3)** | **34.1 (7.6)** | **<0.001** |
| **HNA-2ᵃ fraction** | **5.0 [1.80, 17.0]** | **0 [0, 0]** | **<0.001** |
| a: Human Non-Mercaptalbumin 1/2, b: Body Mass Index, c: Sequential Organ Failure Assesment, d: Simplified Acute Physiology Score, e: Interleukin Blocking therapy (eg. Tocilizumab), f: Low Molecular Weight Heparin, g: Human Mercaptalbumin. * Values from the first 24 hours after ICU admission. Normally distributed variables are presented as Mean (SD), variables with a skewed deviation as Median [Min, Max] | | | |

**FIGURE S1**


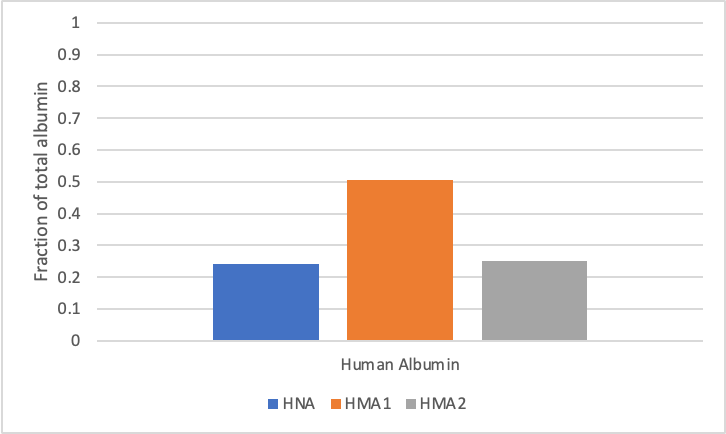


Figure S1: Oxidized albumin fractions in albumin infusate (n=1). HMA = Human Mercaptalbumin, HNA = Human Non-Mercaptalbumin.
